# A major role for Tau in neuronal DNA and RNA protection *in vivo* under physiological and hyperthermic conditions

**DOI:** 10.3389/fncel.2014.00084

**Published:** 2014-03-18

**Authors:** Marie Violet, Lucie Delattre, Meryem Tardivel, Audrey Sultan, Alban Chauderlier, Raphaelle Caillierez, Smail Talahari, Fabrice Nesslany, Bruno Lefebvre, Eliette Bonnefoy, Luc Buée, Marie-Christine Galas

**Affiliations:** ^1^Inserm UMR837, Alzheimer and TauopathiesLille, France; ^2^Jean Pierre Aubert Research Centre, Faculté de Médecine-Pôle Recherche, Institut de Médecine Prédictive et de Recherche Thérapeutique, Université Droıt et Santé de Lille, CHU-LilleLille, France; ^3^Laboratoire de Toxicologie Génétique, Institut Pasteur de LilleLille, France; ^4^CNRS FRE 3235, Génétique Moléculaire et Défense AntiviraleParis, France

**Keywords:** Tau, oxidative stress, hyperthermia, DNA damage, RNA damage, γ-H2AX, DNA repair

## Abstract

Nucleic acid protection is a substantial challenge for neurons, which are continuously exposed to oxidative stress in the brain. Neurons require powerful mechanisms to protect DNA and RNA integrity and ensure their functionality and longevity. Beside its well known role in microtubule dynamics, we recently discovered that Tau is also a key nuclear player in the protection of neuronal genomic DNA integrity under reactive oxygen species (ROS)-inducing heat stress (HS) conditions in primary neuronal cultures. In this report, we analyzed the capacity of Tau to protect neuronal DNA integrity *in vivo* in adult mice under physiological and HS conditions. We designed an *in vivo* mouse model of hyperthermia/HS to induce a transient increase in ROS production in the brain. Comet and Terminal deoxyribonucleotidyltransferase-mediated deoxyuridine triphosphate nick end labeling (TUNEL) assays demonstrated that Tau protected genomic DNA in adult cortical and hippocampal neurons *in vivo* under physiological conditions in wild-type (WT) and Tau-deficient (KO-Tau) mice. HS increased DNA breaks in KO-Tau neurons. Notably, KO-Tau hippocampal neurons in the CA1 subfield restored DNA integrity after HS more weakly than the dentate gyrus (DG) neurons. The formation of phosphorylated histone H2AX foci, a double-strand break marker, was observed in KO-Tau neurons only after HS, indicating that Tau deletion did not trigger similar DNA damage under physiological or HS conditions. Moreover, genomic DNA and cytoplasmic and nuclear RNA integrity were altered under HS in hippocampal neurons exhibiting Tau deficiency, which suggests that Tau also modulates RNA metabolism. Our results suggest that Tau alterations lead to a loss of its nucleic acid safeguarding functions and participate in the accumulation of DNA and RNA oxidative damage observed in the Alzheimer’s disease (AD) brain.

## Introduction

Altered DNA and RNA integrity is particularly harmful in differentiated neurons. Non-repaired nucleic acids trigger transcriptional and translational deregulation, which leads to reduced protein synthesis, protein mutation, the production of truncated proteins and genomic instability. Oxidative stress generates a wide range of nucleic acid lesions including base modifications, deletions and strand breaks. Neurons in the brain continuously face the harmful effects of oxidative stress due to high oxygen consumption. Therefore, the preservation of nucleic acid integrity from oxidative damage is essential to maintain neuronal functionality and ensure their longevity (Englander and Ma, 2006; Chen et al., 2007; Englander, 2008; Mantha et al., 2013). To decipher the defense mechanisms involved in the protection of neuronal DNA integrity in normal brain is crucial to understand DNA alteration observed in neurodegenerative diseases (Brasnjevic et al., 2008; Coppedè and Migliore, 2009).

Tau plays a well-known role in microtubule assembly and stabilization. It has recently been shown that Tau functions as an essential nuclear player in the protection of neuronal genomic DNA integrity under reactive oxygen species (ROS)-producing heat stress (HS) in primary neuronal cultures (Sultan et al., 2011). We observed that oxidative stress and HS in wild-type (WT) neurons led to Tau nuclear accumulation, which protected DNA integrity from HS-induced damage (Sultan et al., 2011). However, the mechanisms responsible for Tau-mediated DNA protection are unknown. DNA protection may be mediated partially through Tau interactions with the A-T-rich DNA minor groove (Sjöberg et al., 2006; Wei et al., 2008; Sultan et al., 2011; Camero et al., 2013); however, a role for Tau in DNA repair mechanisms cannot be excluded.

As this new and major DNA protective role of nuclear Tau has been described in primary neuronal cultures (Sultan et al., 2011). the major aim of this work was to analyze the capacity of Tau to protect neuronal DNA integrity *in vivo* in adult mice under physiological and HS conditions to overcome any potential artifactual effects related to the embryonic origin of cultured neurons.

Using a novel *in vivo* mouse model of transient hyperthermia/HS, this study demonstrated the efficiency of the DNA protective function of Tau in neurons *in vivo*. We showed that Tau was indispensable for the protection of neuronal DNA integrity in the cortex and hippocampus of adult mice under physiological and HS conditions. Tau deletion did not trigger similar DNA damage under physiological and HS conditions. Tau was involved in the DNA double-strand break repair process specifically under HS. Notably, hippocampal neurons in the CA1 subfield showed a reduced ability to restore DNA integrity after HS than neurons in the dentate gyrus (DG). Surprisingly, our data obtained indicated that Tau deficiency altered the integrity of genomic DNA and cytoplasmic and nuclear RNA, suggesting that Tau could protect both RNA and DNA.

## Materials and methods

### Animals

Seven month-old homozygous female KO-Tau mice (Tucker et al., 2001) and littermate WT mice were used to assess the role of Tau in DNA protection in aged mice. All animals were maintained in standard animal cages under conventional laboratory conditions (12 h/12 h light/dark cycle, 22°C), with *ad libitum* access to food and water. The animals were maintained in compliance with institutional protocols and all animal experiments were performed in compliance with, and following the approval of the local Animal Resources Committee (CEEA 342012 on December 12, 2012), standards for the care and use of laboratory animals, and the French and European Community guidelines. Three different mice have been used in each group for all experiments.

### *In vivo* hyperthermia model

We designed an *in vivo* mouse model of transient hyperthermic stress based on the rat model described previously by Papasozomenos (1996). The mice were anesthetized using xylazine (20 mg/kg) and ketamine (100 mg/kg) and maintained in a 37°C environment for 30 min to avoid anesthesia related hypothermia and Tau hyperphosphorylation as previously described (Planel et al., 2007). The mice were then maintained at 37°C (control (C) group) or heat stressed (HS group) by being placed in an incubator containing ambient air heated to 44°C for 20 min. The rectal temperature of the mice was monitored every 10 min and did not exceed 41°C. In the (HS+24H) group, mice were subjected to HS during 20 min and then returned to room temperature during 24 h.

### Oxidative stress-induced protein damage

Protein oxidation was analyzed using an OxyIHC oxidative stress-detection kit (Millipore) according to the manufacturer’s directions. Protein carbonyl groups generated by oxidative stress were visualized using immunolabeling after reaction with 2,4-dinitrophenylhydrazine (DNPH).

### Tissue collection for immunoblotting and confocal microscopy

The mice were euthanized through cervical dislocation, and their brains were rapidly removed. One hemisphere of each brain was post-fixed for 24 h in 4% paraformaldehyde and embedded in paraffin. The hippocampus and cortex were dissected from the other hemisphere and used for biochemical analyses.

### Mouse brain cytoplasmic and nuclear fractionation

Mouse tissues were harvested in ice-cold buffer A (10 mM HEPES, pH 7.9, 1.5 mM MgCl_2_, 10 mM KCl, 0.15% NP-40) supplemented with protease inhibitors (Complete Mini-Roche) and phosphatase inhibitors (125 nM okadaic acid and 1 mM orthovanadate). The tissues were mechanically homogenized using a 50-ml all-glass homogenizer on ice and centrifuged at 100 g for 1 min. The supernatant was collected, and a second homogenization was conducted. The supernatant was collected as the cytoplasmic fraction after centrifugation at 1000 g for 10 min. The pelleted nuclei were washed three times and lysed in ice-cold radioimmunoprecipitation assay (RIPA) buffer containing protease inhibitors (Complete Mini-Roche) and phosphatase inhibitors (125 nM okadaic acid and 1 mM orthovanadate). The samples were sonicated and centrifuged at 12000 g at 4°C for 20 min to yield the supernatant as the nuclear fraction. The protein concentrations were determined using a bicinchoninic acid assay (BCA) kit. Lamin B and synaptophysin (SYP) were used as specific nuclear and cytoplasmic markers, respectively.

### Electrophoresis and immunoblotting

Electrophoresis and immunoblotting were performed as described previously (Sultan et al., 2011) using a Tau C-terminal antibody as described previously (Galas et al., 2006). The results are expressed as the mean ± S.E.M. of three different mice. ImageJ software was used for quantification.

### *In vivo* comet assay

The alkaline *in vivo* comet assay was specifically developed in the cortex for this project. The mouse cortices were dissected and mechanical disaggregation of each tissue was performed by using the Medimachine® system (Becton Dickinson). A small piece of cortex was inserted into a Medicon (i.e., a disposable chamber containing an immobile stainless steel screen allowing for efficient cutting) with approximately 1.0 mL of PBS buffer. The Medicon was thus inserted into the Medimachine® which was then run for 5 s. Once the tissue was processed, the cell suspension was recovered and viability was assessed using the trypan blue exclusion method. Cell viability was assessed using the trypan blue exclusion method. The comet assay was performed as described previously (Sultan et al., 2011). The Olive tail moment (OTM; Olive et al., 1990) was used to evaluate DNA damage. The OTM, expressed in arbitrary units, is calculated by multiplying the percent of DNA fluorescence in the tail by the length of the tail in micrometers. The tail length is measured between the edge of comet head and the end of the comet tail. A major advantage of using the OTM as an index of DNA damage is that both the amount of damaged DNA and the distance of migration of the genetic material in the tail are represented by a single number.

### Terminal deoxyribonucleotidyltransferase-mediated deoxyuridine triphosphate nick end labeling (TUNEL) staining

Terminal deoxyribonucleotidyltransferase-mediated deoxyuridine triphosphate nick end labeling (TUNEL) staining was conducted on tissue slices using the TUNEL Apoptosis Detection Kit (Millipore) according to the manufacturer’s instructions. Tissue slices were pre-treated with low concentration of DNAse (1 µg/mL during 1 h) to perform positive controls. The TUNEL assay is often used to detect late apoptosis-induced DNA breaks; however, it also detects accessible 3′-hydroxyl (3′-OH) groups that are generated from DNA single- or double-strand breaks (DSB) under non-apoptotic conditions (Liu et al., 2005).

### DNAse and RNAse treatments

Brain slices from heat-stressed KO-Tau mice were incubated with DNAse-free RNAse (0.5 mg/mL, 3 h, Roche), RNAse-free DNAse (0.2 mg/mL, 3 h, Millipore #17-141 h) or a mixture of DNAse/RNAse prior to the TUNEL assay.

### Immunofluorescence

Sagittal (5 µM) brain slices were deparaffinized and unmasked using citrate buffer (3.75 mM acid citrate, 2.5 mM disodium phosphate, pH 6) for 10 min in a domestic microwave. The slices were submerged for 1 h in 1% horse serum (Vector Laboratories), and the primary antibodies were incubated overnight at 4°C in the presence of PBS-0.2% Triton using the following primary antibodies: total Tau (Tau CTer) and Tau1 antibodies (Galas et al., 2006) and the phospho-histone H2A.X (Ser 139) antibody from Millipore.

These antibodies were revealed via secondary antibodies coupled to Alexa 488 or 568 (Life Technologies). The sections were counterstained and mounted with Vectashield/DAPI (Vector Laboratories). 4′,6-diamidino-2-phenylindole (DAPI) was used as a chromatin counterstain.

### Imaging systems and immunofluorescence quantification

Mouse hippocampal sections were acquired using an LSM 710 confocal laser-scanning microscope (Carl Zeiss). The confocal microscope was equipped with a 488-nm Argon laser, 561-nm diode-pumped solid-state laser and a 405-nm ultraviolet laser. The images were acquired using an oil 40x Plan-NEOFLUAR objective (1.3 NA) and an oil 63X Plan-APOCHROMAT objective (1.4 NA). All recordings were performed using the appropriate sampling frequency (8 bits, 1024-1024 images and a line average of 4). Serial sections from the three-dimensional reconstruction were acquired using Z-steps of 0.2 µm.

Images is an array of pixel and each pixel contains information about the different light intensity or color. This information is encoded in grid as a gray level. The gray values or gray scale describe the fluorescence intensity of every pixel. Acquisitions in confocal microscope are executed in 8 bits, therefore the different values of gray level of a pixel that can take are from 0 to 255 levels (0 no signal and 255 maximum signal).

**Figure 1:**
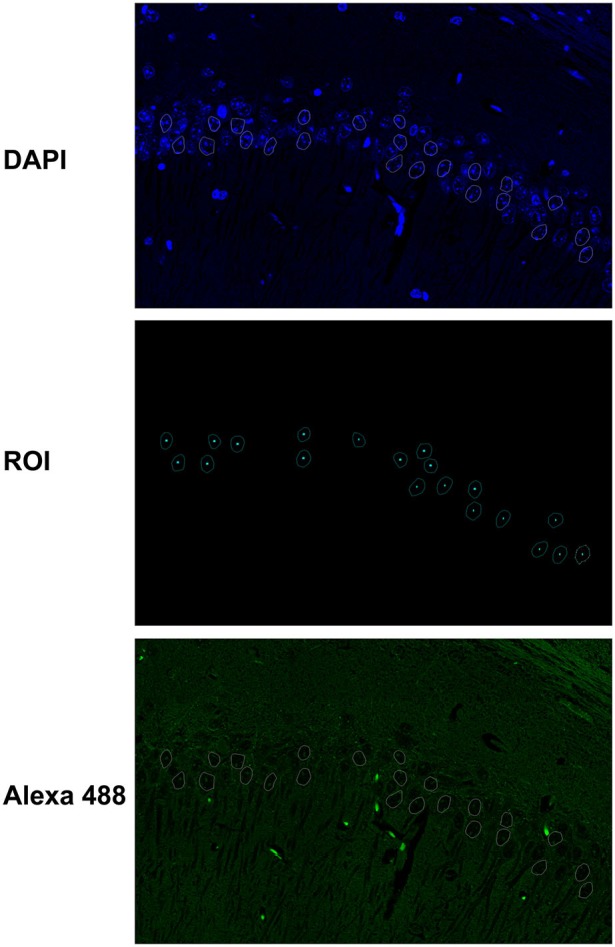
**Fluorescence quantification in the nuclei of hippocampal neurons**. The elliptical selection tool of ImageJ was used to mark 20–30 representative nuclei based on DAPI staining. The immunofluorescence quantifications from the nuclear mean intensity fluorescence are expressed in gray values, and image analyses of the raw data were obtained with ImageJ and ZEN software programs.

The elliptical selection tool of ImageJ marked 20–30 representative nuclei based on DAPI staining (Figure 1). All immunofluorescence quantifications from the nuclear mean intensity fluorescence are expressed in gray values, and image analyses of the raw data were obtained using ImageJ (http://rsb.info.nih.gov.gate2.inist.fr/ij/, NIH, USA) and ZEN (Carl Zeiss) software programs. The results are expressed as the mean ± S.D. of the gray value from 20–30 different nuclei or at least three different areas of the CA1 cell layer.

### Statistics

Student’s *t*-test (BiostaTGV software, Jussieu, France) was used to determine the significance (*p*-value) between groups for immunoblotting and immunofluorescence analysis. A *p*-value < 0.05 was considered to indicate a significant difference.

## Results

### Tau protects neuronal DNA integrity in an *in vivo* mouse model under physiological and hyperthermic conditions

We have shown previously that nuclear Tau protects DNA integrity in primary neuronal cultures of embryonic origin (Sultan et al., 2011). We designed an *in vivo* mouse model of transient hyperthermia/HS that induced ROS production in the cortex and the hippocampus, a region in the mouse brain particularly sensitive to oxidative stress, to investigate the physiological relevance of the DNA-protective function of Tau.

**Figure 2:**
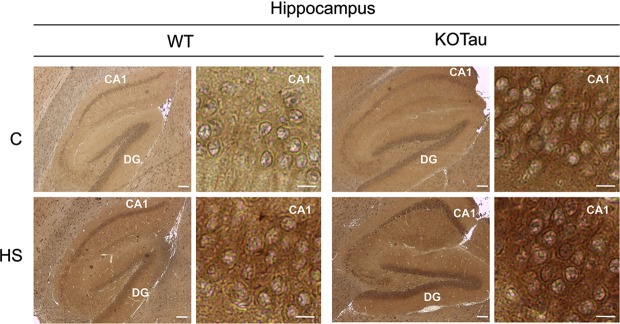
**Hyperthermia generates oxidative stress in WT and KO-Tau hippocampi**. Protein carbonyl groups generated by oxidative stress were visualized using immunolabeling after reaction with 2,4-dinitrophenylhydrazine (DNPH) in sagittal sections of the hippocampus from WT and KO-Tau mice under control (C) or heat stress (HS) conditions. HS increased DNPH staining in WT and KO-Tau sections. *Scale bars* indicate 200 or 10 µm (zoom).

The ability of HS to induce oxidative stress (Flanagan et al., 1998) in the cells of 7-month (7 m) WT and KO-Tau mice was qualitatively analyzed through the immunohistochemical detection of carbonyl groups added to proteins (Figure 2). HS induced an increase of the carbonyl immunolabeling both in WT and KO-Tau cells in sagittal hippocampal sections.

**Figure 3:**
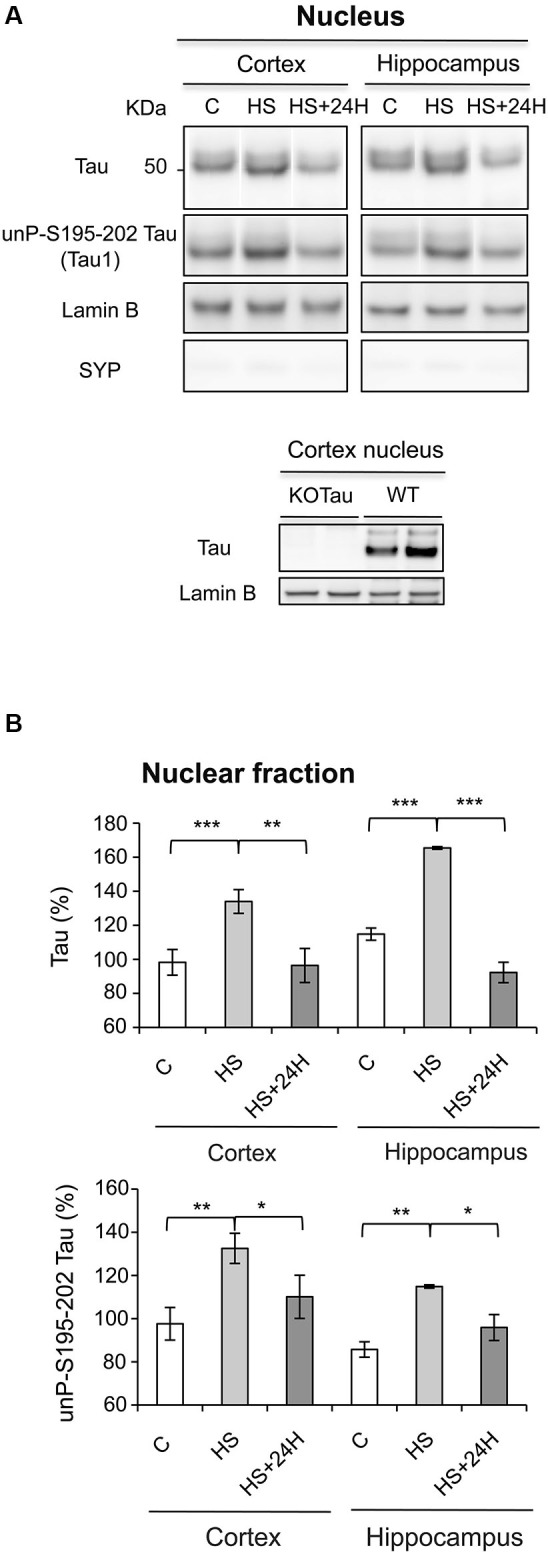
**Nuclear Tau protects genomic DNA integrity from hyperthermia-induced damage. (A)** Nuclear extracts from the cortex and hippocampus of WT mice in the control (C) condition, after HS or after a 24-h recovery after HS (HS+24 h) were analyzed using immunoblotting for Tau independent of phosphorylation (Tau) and Tau unphosphorylated at epitope S195-202 (Tau1). Lamin B and synaptophysin (SYP) were used as specific nuclear and cytoplasmic markers, respectively. **(B)** Densitometric analysis of Tau (normalized to lamin B) and Tau1 (normalized to total Tau) revealed an increase in Tau protein dephosphorylated at epitope S195-202 in the nuclei of neurons under HS. 24 h of recovery restored basal nuclear Tau levels. The data shown are the mean ± S.D. of three different mice. *** *p* < 0.001; ** *p* < 0.01; * *p* < 0.05.

The data obtained after Western blot analysis indicated that Tau was present in the nuclei of neurons from the cortex and hippocampus of WT mice under physiological conditions (Figures 3A, B), which is consistent with previous results in neuronal cultures (Sultan et al., 2011). HS increased Tau nuclear localization, also as observed previously in neuronal cultures (Sultan et al., 2011). The HS-induced nuclear accumulation of Tau in both regions was reversible. Tau in cortical and hippocampal nuclear extracts from WT mice was positively labeled with Tau1 antibody in physiological and HS conditions showing that nuclear Tau was predominantly dephosphorylated at epitope Ser195-202 (Figures 3A, B), as previously described in cultured neurons (Sultan et al., 2011).

**Figure 4:**
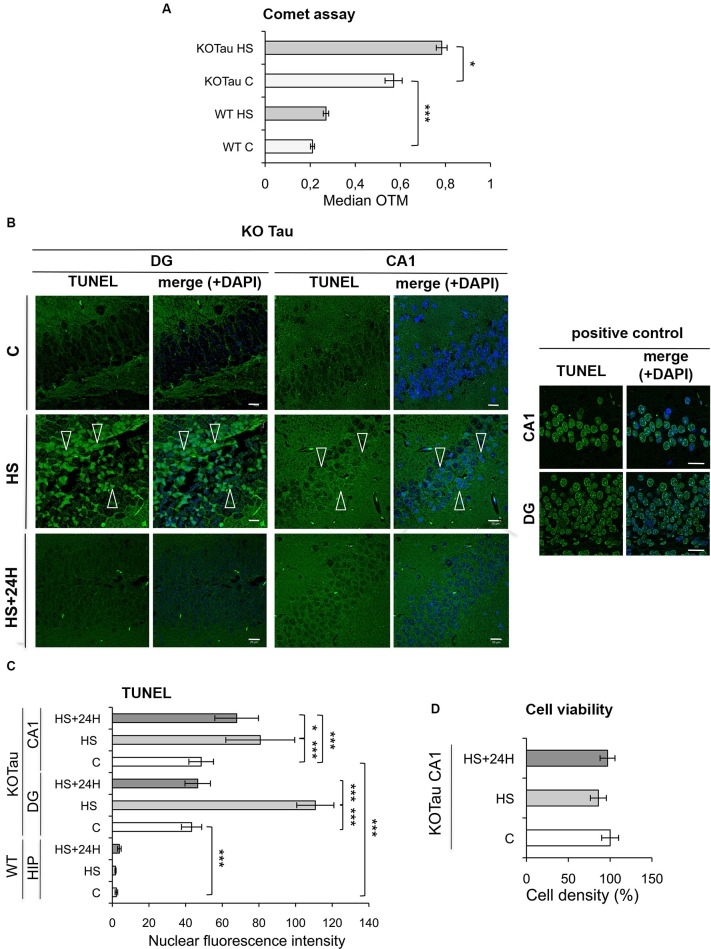
**Hyperthermia increases non-cytotoxic nucleic acid damage selectively in KO-Tau neurons. (A)** The effect of Tau deficiency on genomic DNA integrity was measured using a Comet assay in control (C) and HS mice. The results are presented as the OTM from WT or KO-Tau cortices under C or HS conditions. Tau deficiency selectively promoted DNA damage accumulation and the majority of fragmentation under C and HS conditions. Each OTM value is the median value of 150–200 cells from three different cortices. *** *p* < 0.001; * *p* < 0.05. **(B)** Representative images of the dentate gyrus (DG) and CA1 sagittal sections from 7-month-old KO-Tau mice subjected to TUNEL assay under C, HS and 24 h recovery after HS (HS+24 h) conditions and analyzed using laser scanning confocal microscopy. Nuclei were detected with DAPI staining. HS induced a strong positive TUNEL staining selectively in DG and CA1 KO-Tau neurons. The arrows indicate TUNEL-positive neurons. As a positive control, DG and CA1 sagittal sections from 7-month-old WT mice in control condition have been pretreated with a low concentration of DNAse to create substrate for the end-labeling reaction. *The scale bars* indicate 20 µm. **(C)** The effect of Tau deficiency on nuclear nucleic acid integrity was detected using the TUNEL assay under C, HS or HS+24 h conditions. The level of gray (0 = black; 255 = white) was quantified within the nuclei (based on DAPI detection) in cells from whole WT hippocampi (HIP) or DG and CA1 subfields from KO-Tau hippocampi. Tau deficiency clearly increased the averaged gray levels in the DG and CA1 regions in C and HS conditions. 24 h after HS, the gray level fully returned to basal levels in the nuclei from KO-Tau DG neurons but only partially decreased in the CA1 neurons, which shows the selective weakness of CA1 neurons compared with DG cells in the removal of HS-induced damage. The data shown are the mean ± S.D. of 20–30 nuclei. *** *p* < 0.001; * *p* < 0.05. **(D)** Quantification of DAPI-stained nuclei did not show significant changes in cell density in 7-month-old CA1 KO-Tau mice after HS or HS+24 h. These data indicate that HS-generated nucleic acid damage did not promote cell death.

The degree of DNA damage was monitored using a single-cell gel electrophoresis (Comet) assay in the cortices of 7 m WT and KO-Tau mice under control and HS conditions to analyze the capacity of Tau to protect neuronal DNA integrity *in vivo*. A highly significant enhancement of the median OTM, which reflects DNA fragmentation, was observed in KO-Tau mice compared with WT mice under physiological conditions (2.5-fold in KO-Tau C vs. WT C, *p* < 0.001), which shows that the Tau protein plays a major role in the protection of DNA integrity *in vivo* in the adult mouse brain (Figure 4A). HS treatment also selectively increased the OTM in KO-Tau mouse cells (1.4-fold in KO-Tau HS vs. KO-Tau C, *p* < 0.05); however, no significant induction of DNA damage was observed after HS treatment in WT mice (Figure 4A).

The TUNEL assay was performed to specifically visualize and quantify DNA breaks *in vivo*. Sagittal hippocampal sections from non-treated or HS-treated WT and KO-Tau mice were subjected to TUNEL assays, and the results were imaged using laser-scanning confocal microscopy (Figure 4B). Nuclear TUNEL fluorescence was specifically quantified in the neurons (Figure 4C) of two distinct hippocampal areas, the DG and the CA1 subfield. A strong and highly significant difference was observed between WT and KO-Tau mice under control and HS conditions, consistent with the Comet assays. TUNEL nuclear intensity was dramatically higher in the DG and CA1 of KO-Tau mice compared with WT mice under control condition (≈20-fold increase in DG and CA1 KO-Tau C vs. WT C, *p* < 0.001). Altogether, these observations support an essential physiological role for Tau in the protection of neuronal DNA integrity. HS significantly increased TUNEL-positive cells in KO-Tau mice compared with control (C) non-treated cells (CA1, 1.6-fold in KO-Tau HS vs. KO-Tau C DG, *p* < 0.001; DG, 2.5-fold in KO-Tau HS vs. KO-Tau C, *p* < 0.001). Only a fraction of the hippocampal neurons were TUNEL-positive after HS, which reflects the heterogeneity of the stress response between neurons (Figure 4B). As a positive control, DG and CA1 sagittal sections from 7-month-old WT mice in control condition were pretreated with a low concentration of DNAse to create substrate for the end-labeling réaction (Figure 4B). The level of gray was quantified within the nuclei in cells from DG and CA1 subfields in the positive control and compared to the averaged gray levels in the DG and CA1 regions in HS condition (DG: 70% in KO-Tau HS vs. positive control; CA1: 100% in KO-Tau HS vs. positive control), showing the potent effect of HS to induce nucleic acid breaks in KO-Tau neurons.

KO-Tau mice were allowed to recover for 24 h at room temperature after HS to investigate the later effects of HS-induced DNA damage. The nuclear TUNEL fluorescence in DG neurons returned to basal levels (*p* < 0.001); however, the nuclear TUNEL fluorescence only partially decreased in CA1 cells (1.2-fold in KO-Tau HS+24 h compared with KO-Tau HS, *p* < 0.05; Figure 4C). These results demonstrate the persistence of DNA damage selectively in CA1 neurons.

No change in cell density was detected (Figure 4D), which demonstrates the absence of HS-induced cell toxicity in KO-Tau mice in our *in vivo* HS model.

### H2AX phosphorylation is induced in KO-Tau neurons only after hyperthermia

**Figure 5:**
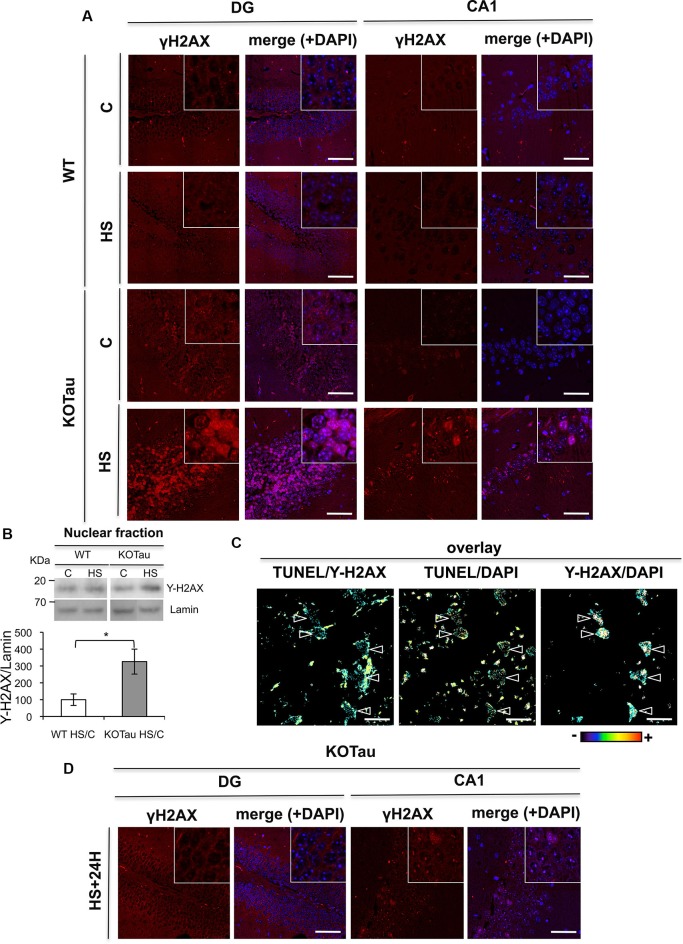
**Tau deletion induces γ-H2AX accumulation under hyperthermia**. H2AX phosphorylation was detected using an anti-γ-H2AX antibody in hippocampal sections from 7 m WT and KO-Tau mice. **(A)** Representative images of sagittal DG and CA1 sections from WT and KO-Tau mice under control (C) or HS conditions labeled for γ-H2AX and analyzed using confocal microscopy are shown. DAPI stained the nuclear chromatin. HS induced a strong increase in γ-H2AX specifically in the KO-Tau hippocampus. *The scale bars* indicate 50 µm. **(B)** Nuclear extracts of the hippocampus from WT and KO-Tau mice in the C or HS condition were analyzed using immunoblotting for γ-H2AX. Lamin B was used as a specific nuclear loading protein. **(C)** Sagittal DG sections from WT and KO-Tau mice were subjected to a TUNEL assay, labeled with γ-H2AX and analyzed using confocal microscopy. Comparisons of TUNEL-γ-H2AX, TUNEL-DAPI and Υ-H2AX-DAPI overlays highlighted the occurrence of double-strand breaks (DBS) only in some nuclei (arrows). *Scale bars* indicate 10 µm. **(D)** Representative images of sagittal DG sections from 7-month-old KO-Tau mice 24 h after HS labeled for γ-H2AX and analyzed using laser scanning confocal microscopy. The nuclei were detected using DAPI staining. Nuclear γ-H2AX labeling returned to control levels in DG neurons, but discrete γ-H2AX foci persisted in the nuclei of CA1 neurons. *The scale bars* indicate 50 µm.

The production of DNA DSB leads to H2AX phosphorylation (γ-H2AX) under normal conditions (Kuo and Yang, 2008), which is necessary to initiate DSB repair. We performed fluorescent immunohistochemical labeling using an anti-γ-H2AX antibody in hippocampal sections from WT and KO-Tau mice before and after HS treatment to investigate DSB formation (Figure 5A). No significant increase in γ-H2AX labeling was observed between 7 m WT and KO-Tau mice under control conditions. However, a strong increase in nuclear γ-H2AX foci was observed in KO-Tau hippocampal (DG and CA1) cells after HS treatment (Figure 5A).

A strong increase in γ-H2AX levels (3.2 fold in HS KO-Tau vs. HS WT, *p* < 0.05) was also specifically observed in lysates from KO-Tau hippocampi after HS treatment (Figure 5B) using immunoblotting. These results indicate that only HS-induced neuronal DNA damage in KO-Tau mice led to γ-H2AX foci production.

γ-H2AX fluorescent labeling and TUNEL assays were performed concomitantly in hippocampal slices of 7 m KO-Tau mice (Figure 5C). HS specifically evoked γ-H2AX foci formation on chromatin in certain TUNEL-positive neurons, as observed in the TUNEL/γ-H2AX, γ-H2AX-DAPI and TUNEL-DAPI overlays.

γ-H2AX labeling in DG cells from KO-Tau mice decreased to basal levels 24 h after HS (Figure 5D), which is similar to the TUNEL staining. This result further confirms the capacity of the DG cells in KO-Tau mice to repair HS-induced DNA damage. In contrast, discrete γ-H2AX foci persisted in the nuclei of CA1 neurons, which indicated the reduced capacity of CA1 neurons to fully restore DNA integrity after HS.

### Hyperthermia alters DNA and RNA integrity in KO-Tau neurons

**Figure 6:**
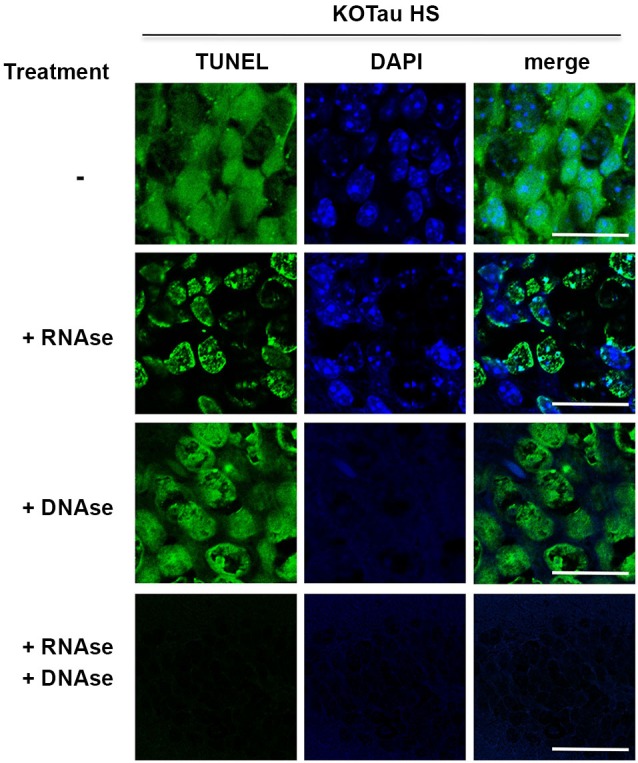
**Hyperthermia causes DNA and RNA damage in Tau-deficient neurons**. Sagittal hippocampus sections from KO-Tau mice subjected to HS were pre-treated or not with DNAse-free RNAse, RNAse-free DNAse or both before TUNEL staining and imaged using laser scanning confocal microscopy. RNAse pre-treatment fully abolished cytoplasmic staining and reduced nuclear TUNEL staining levels. DNAse pre-treatment fully abolished DAPI staining and reduced nuclear TUNEL staining. These data demonstrated that Tau deficiency induced nuclear DNA and cytoplasmic/nuclear RNA damage under HS.

The TUNEL-positive staining described in Figure 3B was also present in the cytoplasm of KO-Tau neurons (Figure 6). Cytoplasmic TUNEL staining was diffuse and, therefore, did not correspond to mitochondrial DNA fragmentation. We hypothesized that the cytoplasmic TUNEL labeling reflected RNA fragmentation because the TUNEL assay is based on fluorochrome labeling of 3′-OH termini after nucleic acid breaks.

Hippocampal sagittal sections from heat-stressed 7 m KO-Tau mice were incubated with high concentrations of DNAse-free RNAse, RNAse-free DNAse or a mixture of DNAse and RNAse to investigate the possibility that the cytoplasmic TUNEL staining corresponded to RNA strand breaks (Figure 6).

Pre-treatment with RNAse fully abolished cytoplasmic TUNEL staining and partially abolished nuclear TUNEL staining, which suggested that the cytoplasmic staining and a portion of the nuclear staining was HS-induced RNA damage in KO-Tau neurons. Pre-treatment with DNAse only partially removed nuclear TUNEL staining despite the complete disappearance of DAPI staining, which indicates complete DNA degradation. This result suggests that nuclear RNA is damaged after HS in KO-Tau neurons. Concomitant pre-treatment with DNAse and RNAse fully abolished cytoplasmic and nuclear TUNEL staining, which confirms the specific alterations of these nucleic acids in stressed KO-Tau neurons. These results suggest that DNA and nuclear and cytoplasmic RNA are damaged after HS in KO-Tau neurons.

## Discussion

### DNA protective function of Tau *in vivo*

The present study demonstrates a novel, major physiological role for nuclear Tau in the protection of neuronal DNA integrity *in vivo* in the adult mouse brain under physiological conditions; the absence of Tau rendered neuronal cells abnormally susceptible to HS-induced DNA damage.

Neurons in the brain encounter recurrent oxidative stress throughout their lifespan. The high basal levels of DNA damage in KO-Tau compared with WT neurons in aged mice under physiological conditions likely reflected a loss of the intrinsic protective function of Tau against chronic endogenous oxidative stress in the brain. It may contribute to the different deficits observed with age in Tau KO mice (Ke et al., 2012).

We designed a novel *in vivo* mouse model of transient hyperthermia/HS as a valuable and useful tool to easily investigate the effects of a transient and acute ROS increase in the whole brain of WT or transgenic mice. This model can be used to delineate the role of Tau in DNA protection under oxidative stress conditions. However, we cannot exclude the possibility that effects other than oxidative stress are involved in hyperthermia (Morano et al., 2012). This model demonstrated that hyperthermia potentiated DNA alterations in the absence of Tau *in vivo*, which reproduces the DNA protective role of Tau under HS conditions in primary neuronal cultures (Sultan et al., 2011).

Altogether these results show that Tau plays an essential role to preserve DNA integrity in adult neurons *in vivo* under physiological and HS conditions.

### Tau modulates DNA double-strand break repair

DNA damage in neurons triggers a cascade of highly potent DNA repair mechanisms to maintain genome integrity (Canugovi et al., 2013). DNA damage responses can induce highly dynamic post-translational modifications of histones that are critical for the DNA repair process (Lukas et al., 2011). DBS, one of the most toxic forms of DNA damage, quickly induce phosphorylation at serine 139 in the C-terminal sequence of histone H2AX (γ-H2AX), which promotes the recruitment of multiple DNA repair factors.

The absence of strong γ-H2AX accumulation in KO-Tau neurons under control conditions notwithstanding the strong increase in neuronal DNA damage in KO-Tau compared with WT mice observed using Comet and TUNEL assays, indicated that the DNA damage accumulation in Tau-deficient mice under physiological conditions differs from the HS-induced damage. This result suggests that chronic oxidative stress primarily induces single breaks rather than DSB or that the DSB repair process is impaired in KO-Tau neurons under physiological conditions.

Conversely, hyperthermia induced a strong and transient increase in γ-H2AX foci selectively in the nuclei of KO-Tau neurons, indicating that Tau deficiency induced the accumulation of non-repaired DNA DSB through altered DNA damage-induced chromatin post-translational modifications. This result suggests that Tau modulates double-strand break DNA repair responses under hyperthermia.

Hyperthermia-induced nucleic acid damage is reversible, which suggests that a Tau deficiency delays, but is not critical for, the DNA repair process. Tau likely plays a modulating rather than an essential role in double-strand break DNA repair mechanisms, and other proteins could compensate for its loss of function.

These results show an essential role for Tau in the control of DNA breaks under physiological conditions in adult neurons *in vivo* and a modulating role in the DNA double-strand break repair process under hyperthermia. Overall, this study suggests an important role of Tau in DNA repair mechanisms although we cannot exclude that Tau may only be involved in DNA protection.

### Differential DNA vulnerability among hippocampal neurons

DNA from hippocampal neurons in the CA1 subfield showed a higher susceptibility to damage than DG neurons 24 h after hyperthermia. This result highlights the relative weakness of KO-Tau CA1 neurons to restore DNA integrity compared with DG neurons.

Selective deficiency in oxidized DNA repair has been reported in CA1 neurons compared to others hippocampal neurons under oxidative stress conditions, hypoxia, ischemia and neurodegeneration in Alzheimer’s disease (AD; Wang and Michaelis, 2010).

Our data suggest that impaired Tau-dependent DNA repair plays a role in the selective vulnerability of CA1 neurons.

### Relationship between Tau and RNA metabolism

The present *in vivo* study suggests that Tau deficiency triggers alterations in RNA integrity in hippocampal neurons under HS.

Indeed, RNA oxidation predominantly leads to strand breaks (Poulsen et al., 2012). Cytoplasmic TUNEL staining has been described, but RNA fragmentation is rarely suggested as a possible cause. Nevertheless, TUNEL protocols often advise the use of RNAse treatment to clear the so-called cytoplasmic background (Zhang et al., 2006).

However, as RNA integrity has been analyzed in an indirect way, further experiments like mass spectrometry would be necessary to confirm the alteration of RNA in KO-Tau mice.

It is generally acknowledged that altered RNA is degraded rather than repaired because very few RNA repair mechanisms have been described in mammalian cells (Aas et al., 2003; Nunomura et al., 2009). Cleaved RNA is particularly harmful for neurons because it can lead to the translation of dysfunctional truncated or mutated proteins. RNA damage accumulation in KO-Tau neurons suggests that Tau plays a role in the RNA quality control process in addition to its previously described genomic DNA protective function. Tau is an RNA-binding protein (Kampers et al., 1996), and Tau may protect RNA partially through direct or indirect interactions. Further experiments are necessary to elucidate the potential role of Tau in RNA metabolism.

## Conclusions

Our data suggest that Tau protection of DNA and RNA integrity plays a key role in nucleic acid integrity under physiological conditions and under ROS-producing stress such as hyperthermia.

Tau is impaired in several devastating neurodegenerative diseases (i.e., tauopathies) such as AD. An increase in oxidative DNA (Brasnjevic et al., 2008; Coppedè and Migliore, 2009; Bradley-Whitman et al., 2014) and RNA (Lovell et al., 2011; Nunomura et al., 2012) damage occurs in a subset of vulnerable neurons that exhibit Tau pathology during the early stages of AD. Therefore, the pathological forms of Tau may have altered nucleic acid protective functions. A loss of Tau-mediated nucleic acid functions may participate in the DNA and RNA damage accumulation observed in tauopathies.

## Author contributions

Marie Violet, Lucie Delattre, Alban Chauderlier, Audrey Sultan and Raphaelle Caillierez performed the *in vivo* HS model, immunohistochemistry, TUNEL assay, Western Blot and cell viability. Meryem Tardivel performed the confocal microscopy analysis and quantification. Smail Talahari performed the Comet assay. Fabrice Nesslany designed and supervised the Comet assay. Luc Buée and Marie-Christine Galas designed and supervised the experiments. Eliette Bonnefoy, Bruno Lefebvre, Luc Buée and Marie-Christine Galas interpreted the data. Marie-Christine Galas wrote the manuscript. Eliette Bonnefoy, Bruno Lefebvre and Luc Buée revised the manuscript critically for important intellectual content. All authors approved the final version of the manuscript and are accountable for all aspects of the work.

## Conflict of interest statement

The authors declare that the research was conducted in the absence of any commercial or financial relationships that could be construed as a potential conflict of interest.
